# A Sensorized Nuss Bar for Patient-Specific Treatment of *Pectus Excavatum*

**DOI:** 10.3390/s141018096

**Published:** 2014-09-29

**Authors:** Stefano Betti, Gastone Ciuti, Leonardo Ricotti, Marco Ghionzoli, Filippo Cavallo, Antonio Messineo, Arianna Menciassi

**Affiliations:** 1 The BioRobotics Institute, Scuola Superiore Sant'Anna, Pontedera (PI)56025, Italy; E-Mails: g.ciuti@sssup.it (G.C.); l.ricotti@sssup.it (L.R.); f.cavallo@sssup.it (F.C.); a.menciassi@sssup.it (A.M.); 2 Department of Pediatric Surgery, Children's Hospital A. Meyer, Florence 50139, Italy; E-Mails: marcoghionzoli@hotmail.com (M.G.); a.messineo@meyer.it (A.M.)

**Keywords:** implantable sensorized device, Nuss technique, *Pectus Excavatum*, pressure/force monitoring system, smart prostheses, thoracic surgery

## Abstract

*Pectus Excavatum* is an anatomical deformation characterized by a depression of the anterior chest wall. Nuss technique (representing the current clinical golden standard) consists in the introduction of a corrective metal bar, in order to raise the sternum in its anatomic natural position. Nowadays, the bar plays purely a mechanical/corrective action and is kept implanted for about three years, supporting up to a maximum force of 250 N. Our study aims at optimizing the procedure of correction, in terms of monitoring the bar effect, minimizing the body response, and facilitating the bar removal. The sensorized Nuss bar prototype inserted in a platform for telemedicine described in this article is able to monitor *in vitro* pressure data variations, with more than 150 discrete measurements during the operating period. This behavior is promising for future clinical applications, in which the device could be exploited to monitor the forces at work, thus, providing a customized therapeutic protocol, which in turn may optimize the period of implant. The sensorized bar was also provided with a polymeric coating, able to influence human dermal fibroblast behavior *in vitro*. This highlights the possibility to minimize, in future *in vivo* applications, tissue fibrotic responses.

## 1. Introduction

*Pectus Excavatum* (PE) represents a depression in the anterior chest wall resulting from a dorsal deviation of the sternum [1]. PE is the most frequent chest deformity in children, affecting 0.4%–1% of the population, with a male to female ratio of 4:1 [2]. Deformities of the chest wall are characterized by a funnel-shaped anterior region and by the reduction of the distance between the sternum and the spinal column. Over the years, there has been a great deal of debate about the possible causes of PE. It seems related to a metabolic defect that leads to weakness of the sternocostal cartilage and matrix disorganization [3]. The deformities may be either evident at birth or become visible during development.

PE, in most instances, has little or no influence on the function of the inner organs; nevertheless, the cosmetic appearance of the patients often leads to psychological impairments that require therapy [1]. Moreover, in the case of pronounced defects, the reduction of thoracic volume may result in reduced cardiovascular and pulmonary functions.

Different surgical and non-surgical treatments have been proposed [1], each characterized by different drawbacks, such as visible scars [4], high invasiveness [5,6], or poor success rates [7]. In 1998, Nuss introduced a minimally invasive technique for the correction of PE, which is, today. The most used therapeutic strategy for the correction of this deformity [8]. The main advantage of Nuss technique arises from the absence of visible scars on the chest, the absence of osteotomy and costal cartilages resection, the limited loss of blood, the ability to correct asymmetries, and the ability to obtain a satisfactory and long-lasting correction. For this procedure, two skin incisions of 30–40 mm in length are made at the right and left middle axillary lines. A metal introducer is inserted intrathoracically on the right costal ridge, and a way through the anterior mediastinum is made, thus, separating the sternum from the pericardium. A tie is attached to the end of the introduced bar and the introducer is then pulled back, allowing the passage and the positioning of the tie in the predetermined intercostal space. The tie is then attached to the final bar so that it can be inserted with the concave side facing forward, guided by the tie, pulled up from the other side. Under thoracoscopy, the retrosternal curve bar is rotated by 180° around its axis so that the concave side faces backwards, thus, pushing the sternum in a ventral direction [9]. The metal bar is connected with one or two stabilizers and attached to the muscles of the chest wall [10].

**Figure 1.**
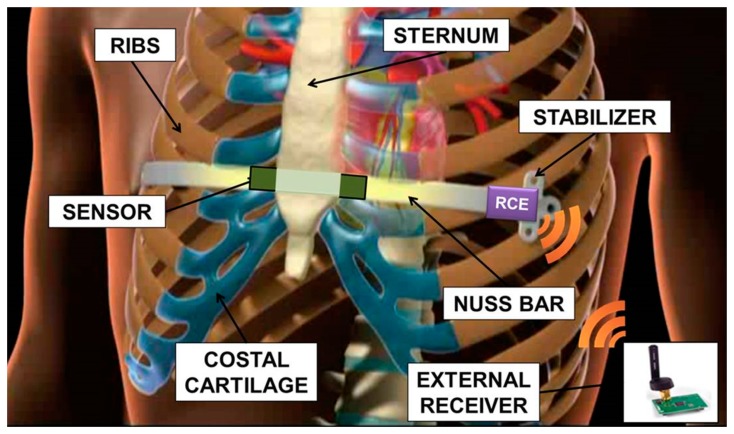
Schematic representation of the sensorized Nuss bar. Remote-controlled electronics allow, through wireless communication, to send the force signal to an external receiver.

The non-absorbable nature of the implant involves a second surgical operation, which usually takes place after three years. Important issues arise from leaving a stainless steel implant in the chest: firstly, during the period of implant, there is evidence of metallic ion diffusion, which may have an impact on health, being that most of the treated patients are adolescents with a long life expectancy [11]. Moreover, long-term stainless steel implants imply an important interaction between biological tissues and the metal device: this often results in an excessive fibrotic reaction. For these reasons the treatment should be limited to the time necessary to achieve a stable correction of the defect. The Nuss technique is mainly carried out on adolescent patients, not exhibiting too fast a growth and evolution of the chest shape in the three years of the implant, but still having a flexible thorax. The sensorization of the Nuss bar, schematically represented in Figure 1 and described in this paper, would allow to monitor the progressive correction of the deformation, in order to determine its trend and to clarify when a complete and lasting correction has been obtained. This introduces the disruptive possibility of a personalized treatment of PE. A decrease in the implant time, compared to the three years currently adopted, brings all the advantages related to a reduced interaction between tissues and bar (thus, assuring a less traumatic removal). It also allows to expand the range of patient ages in which it is possible to apply the corrective Nuss technique, e.g., those characterized by rapid physical development, thus, reducing the complications associated with the presence of an implant for a long period of time.

## 2. System Overview

**Figure 2.**
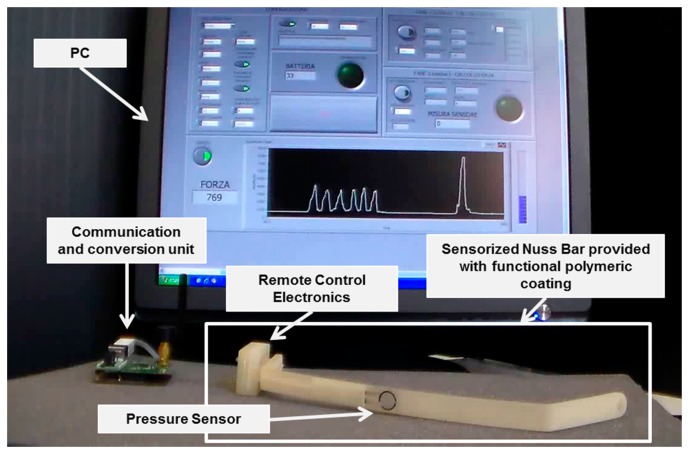
Prototype of the system: A prototype of the sensorized Nuss bar was developed in a plastic resin by using a 3D printer (3DSYSTEM PROJET HD 3000), provided with key components, enabling the management and transmission of pressure data. Both the sensorized Nuss bar structure and the sensor were covered by a micrometric polymeric coating.

The targeted system was a sensorized Nuss bar, provided with an engineered polymeric coating, able to monitor the force exerted by the sternum on the prosthesis and to provide the patient and the clinicians with pressure data. Figure 2 shows a mockup of the system: it was based on a custom-made Nuss bar with an embedded pressure sensor and provided with a thin polymeric coating, remote-controlled electronics (RCE), a communication and conversion unit, and a personal computer (PC), provided with a dedicated interface.

**Figure 3.**
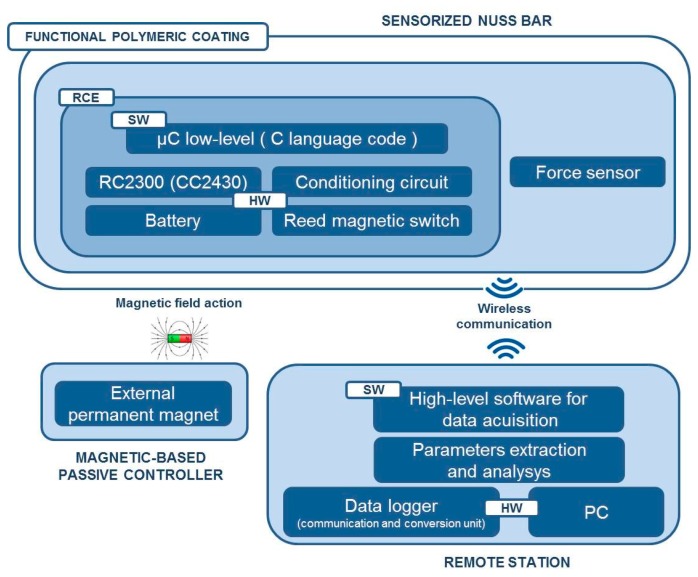
System architecture, components, and communication.

Figure 3 shows the overall system architecture and the single components. The sensorized Nuss bar was based on a miniaturized integrated electronic circuit, constituted by a thin force sensor integrated in the bar, a conditioning circuit, a microcontroller, a rechargeable battery, and a reed magnetic switch acting as the trigger for the measurement process and data collection. A C language-based firmware was developed to acquire data from the force sensor and to send them, via wireless communication, to a remote data logger. The sensorized Nuss bar was provided with a functional polymeric coating, aiming at minimizing the adverse reactions of the organism to the implant.

The activation and deactivation of force data acquisition was achieved by a magnetic-based trigger: a permanent magnet, integrated in a credit card-shaped case, was used to activate the system in a wireless fashion, by placing the card in the proximity of the sensorized Nuss bar (*in vivo*, it is supposed to be placed on the patient's skin) and, thus, changing the status of the reed magnetic switch.

A detailed description of the single modules of the system (*i.e.*, mechanical system design, electronic and software implementation, and functional coating development and characterization) is provided in the next sections.

## 3. Mechanical Design and Finite Element Method (FEM) Simulations

The first step consisted in the mechanical design of a Nuss bar, integrating the sensing element and the polymeric coating. The need for integrating these two components in a conventional Nuss bar reduced the space available for the structural metal component (especially in terms of thickness). The currently used metal bars (made of steel, AISI 316L) have sizes in a range of 178–458 mm in length, 2.5–3.5 mm in thickness, and about 12.7 mm in width, depending on the anatomical features of the thorax [12]. In particular, due to the presence of a force sensor (thickness of 0.2 mm) and of a polymeric coating, of which the thickness is well below 100 μm, the thickness of the Nuss bar metal component was reduced from 2.5 to 2.3 mm. To evaluate if such a modification could compromise the overall bar mechanical stability, we performed finite element model (FEM) analyses, by evaluating stress, strain, and deformation in response to an applied pressure. Three-dimensional Computer Aided Design (CAD) elements were designed (PRO/ENGINEER PTC, Needham, MA, USA), then imported and processed by ANSYS FEM simulation software (Canonsburg, PA, USA, static structural simulation with element type SOLID186). In the clinical setting, the bar shape is manually modeled by the medical doctor, based on X-ray images of the patient's chest. An appropriate bending instrument is used immediately before the surgical intervention. Being unrealistic to consider a single curvature of the bar, simulations were performed by considering specifications provided by a medical team of the Children's Pediatric Hospital A. Meyer for 14 typical configurations of Nuss bars, related to different chest dimensions (configurations A to G short and long, respectively—Detailes are reported in [13]).

According to the study conducted by Webber *et al.* [14], the maximum force that the sternum can exert on the bar is about 250 N. A bar made of AISI 316L and a target load of 250 N were considered in the simulations. As a consequence, a yield tensile strength (YTS) of 590 MPa [15] was considered as the target value the Nuss bar had to not overcome in our analysis when the target load was applied.

**Figure 4.**
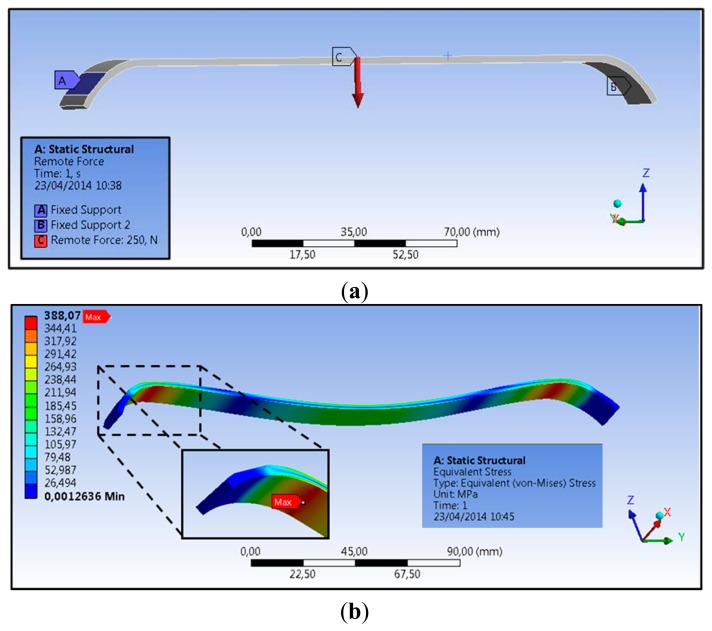
(**a**) CAD representation of Nuss bar with an applied force of 250 N (red arrow—C) and the two “fixed support” constraints (blue areas—A, B); (**b**) Stress distribution on the bar. The area subjected to the maximum stress is shown in the inset. Nuss bar D long configuration is represented in the figure as an example of the 14 simulations.

The applied force vector and the mechanical constraints considered on the Nuss bar are represented in Figure 4a (for a specific bar type, D long configuration). Two specific constraints, *i.e.*, fixed support, were placed on the bar (blue areas—A and B), replicating the contacts between the coast and the top/bottom faces of the bar. The applied force, representing the reaction of the sternum on the bar, is placed centrally on the top surface (red arrow—C). It is assumed that this is, realistically, the only significant force acting on the entire system.

**Figure 5.**
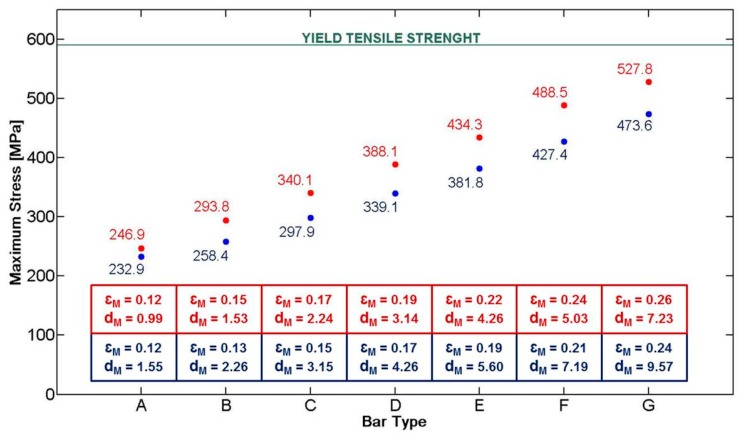
Graph showing the maximum stress values in correspondence to different bar types, loaded with a force of 250 N. The set of bars named “short” is represented in red, while the “long” set in blue. The values of maximum strain (ε_M_[%]) and maximum deformation (d_M_ [mm]) are reported in the boxes, for each bar type.

The stress distribution on the Nuss bar is reported in Figure 4b; similar distributions were obtained also for the other Nuss bar types. As expected, the maximum stresses and strains are concentrated at the pre-set curvature of the bar, while the maximum deformation is evidenced at the force application point. For all the configurations, *i.e.*, from A to G short and long, the stresses remain below the AISI 316L YTS, thus guaranteeing Nuss bar mechanical stability against the imposed target load (Figure 5). A maximum deformation of 9.5 mm was discovered in the worst case (*i.e.*, G long configuration); it was deemed not compromising the surgical outcome.

## 4. Electronic System Design, Sensor Characterization and Software Implementation

### 4.1. Electronic System Design

**Figure 6.**
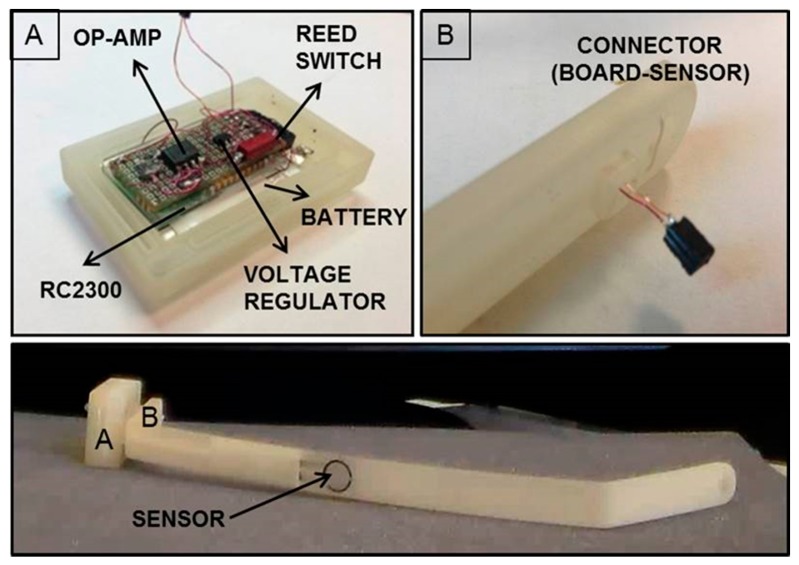
(**A**): RC2300, battery and conditioning circuit in rectangular-shaped rigid protective package. The figure shows the custom conditioning-circuit board. The electrical circuit conditioning is composed mainly of an operational amplifier in non-inverting configuration; (**B**): The connector between the sensor and the electronics. Bottom: Entire implant prototype.

The miniaturized integrated electronic circuit, based on the 8051 family embedded microcontroller and provided with a wireless additional communication module, was designed and developed by the authors, integrating off-the-shelf components in order to record and store data from the installed force sensor. The board design and specifications were critically investigated due to the dimension constraints; the electronic board prototype, including an IEEE 802.15.4 compliant transceiver, is 35 mm in length and 20 mm in width, and has an overall thickness of about 10 mm with all the components assembled. The miniaturized integrated electronic circuit comprises an RC2300 transceiver (Radiocrafts, Oslo, Norway) module embedding a CC2430 (Texas Instruments, Dallas, TX, USA) system-on-chip core component, selected for its low power consumption and suitable dimension, and comprising a wireless module over 2.4 GHz ZigBee compliant radio hardware (Figure 6).

The force-sensitive resistor (FSR) sensor FlexiForce A201 (Tekscan, Inc., South Boston, MA, USA) was used as the sensing element, because it was one of the thinnest sensors on the market, with a thickness of about 0.2 mm; the overall platform was designed to be able to interface different FSR sensors with, also, multisensitive areas (*i.e.*, up to eight sensors can be connected to the electronic board). The force data (*i.e.*, as the variation of resistance) are converted into a voltage variation through a conditioning circuit, consisting of an operational amplifier in a non-inverting configuration, and later sent to the Analog to Digital Converter (ADC) input of the microcontroller for digital conversion and management.

**Figure 7.**
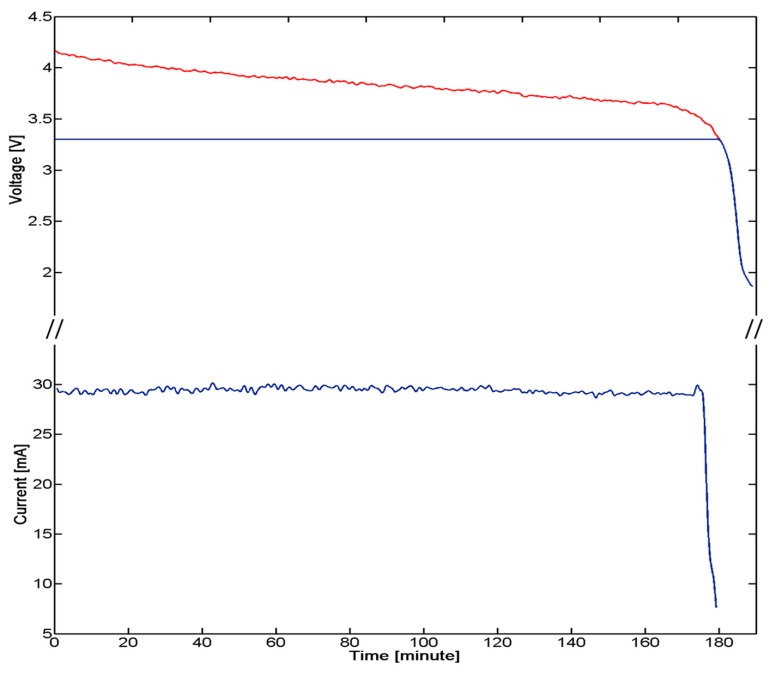
A test of battery life. (**Top**) the trend of the voltage across the battery, over time, is plotted in red, while the actual supply voltage of the circuit, due to the voltage regulator, is plotted in blue; (**Bottom**) the instantaneous current consumed by the system is plotted in blue. The measurement of the pressure is guaranteed, from the device, for at least 170 min in configuration ON.

A critical aspect of the implanted device derives by the need of having a low power consuming, for ensuring the functioning of the system for an appropriate time defined by the requirements of the system and procedure itself. A strategy for power control consisted of the integration of a reed switch (CT05-3050-G1, Coto Technology, Sunnyvale, CA, USA) used as a switch of the electronic board and controlled by the magnetic-based trigger card; in addition, a voltage regulator was installed for peripheral power control (TPS78230DDC, Texas Instrument, Dallas, TX, USA). A graph of the power consumption of the device is represented in Figure 7: Data were collected with a digital oscilloscope (InfiniiVision 7034B, Agilent Technologies, Santa Clara, CA, USA).

The overall system is supplied by an integrated 100 mAh battery (3.7 V Li-Po PGEB242030-Power Stream, UT, USA). An average consumption of 29 mA for over 170 min was experimentally assessed. The user, approaching a small magnet (represented by the magnetic-based trigger) to the patient's chest, requires a measure of the pressure. The magnet, acting on the Reed Switch embedded in the miniaturized integrated electronic circuit, turns on the entire system. Considering an estimated time for measurement, in the order of one minute for each acquisition, a monitoring protocol of the correction of PE with at least 170 measurements during the treatment period can be assumed.

In order to properly interface the wireless electronic board to the human-machine interface (HMI), a second CC2430-based module was exploited and connected to the PC through a Universal Serial Bus (USB) port, through an off-the-shell USB/serial Universal Asynchronous Receiver-Transmitter converter development module for the FT232R IC device (UM232R, Future Technology Devices International Ltd., Glasgow, UK) (communication and conversion unit—Figure 2).

### 4.2. FSR Sensor Characterization

**Figure 8.**
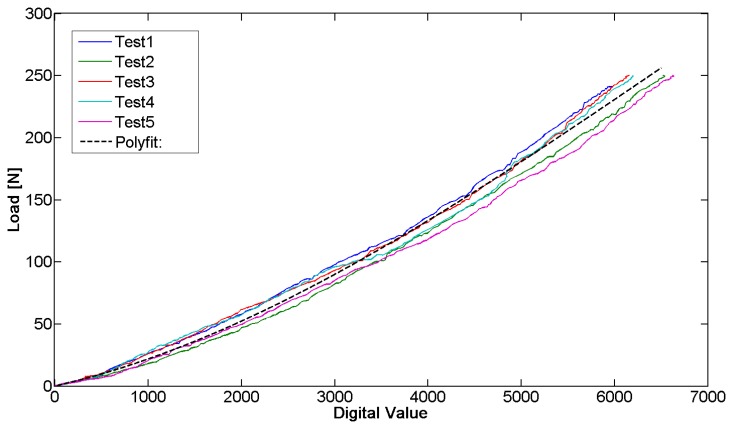
A sample of the calibration curves obtained from five tests performed on one of the five different FlexiForce sensors. The dotted line represents the polynomial interpolation of degree 3 that fits the data in a least squares sense. This curve was then used to convert the digital value provided by the system in the corresponding force value during the system validation.

The sensor behavior was characterized by a mechanical testing system (INSTRON 4464, Boston, MA, USA), exploited to perform compression and progressive reduction of the load on the sensor with a load cell (maximum value ± 1 KN). Our aim was to obtain the correlation between the sensor output and the acting force. The tests allowed deriving the force/signal relation and assessing the repeatability of the measurement. The sensor has been positioned on the metal bar and compression tests were subsequently performed on it. An end-effector was custom-designed to be interfaced with the load cell of the INSTRON machine and to focus the pressure on the sensitive area of the bar. The sensor was then loaded with a force equal to 250 N for one minute. The load cell was then elevated from the bar at a speed of 0.5 mm/min, until zero load was reached. Throughout the calibration phase, the sensor output signal, conditioned by means of the dedicated electronics, was synchronized with the force signal deriving from the load cell by means of a LabVIEW routine. It was then used to extrapolate the calibration curve. This procedure was repeated five times for each sensor and five different FlexiForce A201 sensors were tested. A typical set of data is represented in Figure 8.

The data sampling, in calibration tests, was performed at 2 Hz. The sampling rate was kept constant through the firmware code for the entire duration of the tests.

The repeatability error calculated between the five tested sensors (43.7%) indicates the need to perform the calibration for each sensor before it is plant. Instead, the repeatability error (13.0%), averaged over five tests carried out on the same sensor, is sufficiently low to ensure the monitoring of the pressure in our specific application. The purpose is to identify the plateau at the end of the phase of decrease and not to have an accurate measurement of the acting pressure. Moreover, the linearity error of the measuring device is equal to 11.2% and the hysteresis that afflicts the measurement system reaches a maximum of 54.8 N. The hysteresis is negligible because the force measured by the sensor in the presented application is only decreasing. The resolution of the sensorized bar, which depends on the sensor technology and electronics, is 1.5 N.

### 4.3. Software Implementation

A software was implemented to handle the required tasks during: (i) data acquisition and managing control; and (ii) communication process. The sensing and communication software, developed in C-language on the IAR Embedded Workbench development platform (IAR Systems, Stockholm, Sweden), was implemented to efficiently sense the acting force and continuously send the acquired values to the data logger at a frequency of 2 Hz.

The communication and conversion unit implements a second C-based software application and acts as a communication bridge between the bar and the PC for data management and message processing.

**Figure 9.**
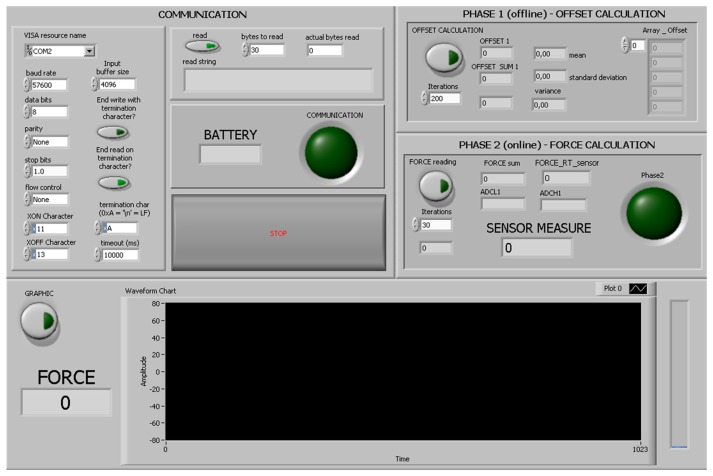
The LabVIEW HMI allows the visualization of the force during capture sessions under the monitoring protocol, the automatic saving of data and creation of a database of the patient, the visualization of the data in real-time, and the monitoring of the power.

The higher-level HMI was developed in LabVIEW (National Instruments, Austin, TX, USA) as a control panel to receive the sensory feedback from the Nuss bar, and to send control commands to the connected peripherals (Figure 9). In particular, the HMI can be employed by the user to select the serial communication options and, once the system is activated with the magnetic-based trigger, to request the force data.

## 5. Engineered Polymeric Coating

### 5.1. Motivation

The use of a polymeric coating on the Nuss bar aims at minimizing the undesirable tissue responses, typical when a metal device is implanted in the body [16].

Independently, on its topography, the presence of a polymeric coating limits the inflammatory response by improving the biocompatibility of the implant [17].

In our case, we used Polylactic Acid (PLA), a widely known biocompatible polymer, largely used for biomedical applications [18]. In addition, the polymer surface topography was properly structured in order to minimize the fibrotic response of the organism and the growth of soft tissues around the implant, thus, reducing the risk of undesired adherences between the bar and the surrounding tissues. This would reduce the difficulties encountered by surgeons during the intervention aiming at removing the bar at the end of the therapy.

It has been demonstrated that a porous surface entails a reduced inflammatory/fibrotic response. Instead, if the surface is smooth, the thickness of the fibrous capsule, which goes to cover the bar, increases during the time of implant [19,20]. A tissue-specific structure concerning the topography of the employed polymeric coating is, thus, needed.

### 5.2. Material and Methods for Coating Development

To fabricate the engineered coating, we used a 2% w/v solution of PLA (MW = 67,000 g/mol, Sigma-Aldrich) in chloroform. PLA's rate of degradation is longer than three years, thus, ensuring its permanence on the bar surface for the entire time of implant. The PLA solution was used to fabricate a thin polymeric film on the bar surface by exploiting three different techniques: (1) spin-assisted deposition; (2) spray by airbrush, and (3) spray by airbrush coupled with salt leaching.

Spin-assisted deposition cannot be considered an easily usable option as the shape of the bar does not allow a simple fabrication of the coating with this technique. However, the samples realized with this methodology were used as controls. A spin coater (WS-650 spin processor, Laurell Technologies Corp., North Wales, PA, USA) was used, by setting 2000 rpm and 30 s as working parameters.

The same PLA solution was inserted in the reservoir of an airbrush (L. Fisher Letraset, France). This instrument allows to obtain a flow of any liquid solution through the use of compressed air, thus, achieving a uniform spray deposition on the target. In our case, we applied a pressure of 2 bar and we sprayed the PLA solution over the sample surface for about 10 s, for each sample. To obtain a more porous and irregular topography, a salt leaching technique was coupled to the spraying. Immediately after the spray deposition of a PLA layer (before its polymerization), by means of the airbrush and by using the same working parameters, the sample surface was uniformly covered with 50 mg of NaCl crystals, characterized by a diameter of 250 μm (Sigma-Aldrich, S7653).

All the sample types were then thermally treated for 60 s at 80°C to ensure a full polymerization of PLA. Then, they were placed in deionized water for 12 h to allow salt dissolution, in the case of NaCl-provided samples.

### 5.3. Characterization of the Thickness and Surface Topography

The different sample types were analyzed in terms of thickness and surface roughness. Thickness was measured through a profilometer. The film thickness was ∼1 μm in the case of the spinned samples and ∼10 μm in the case of the sprayed samples.

**Figure 10.**
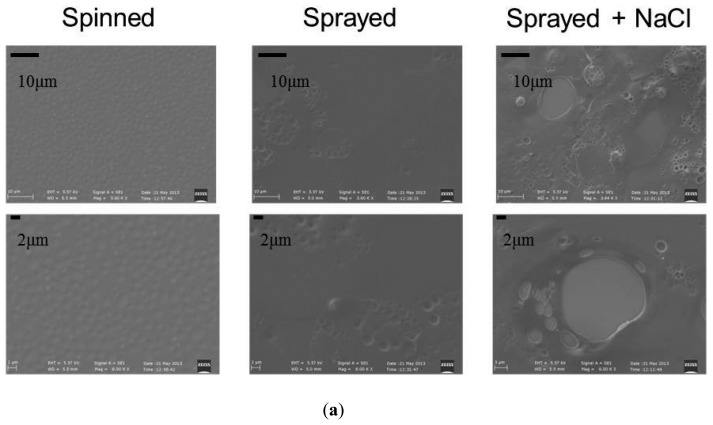

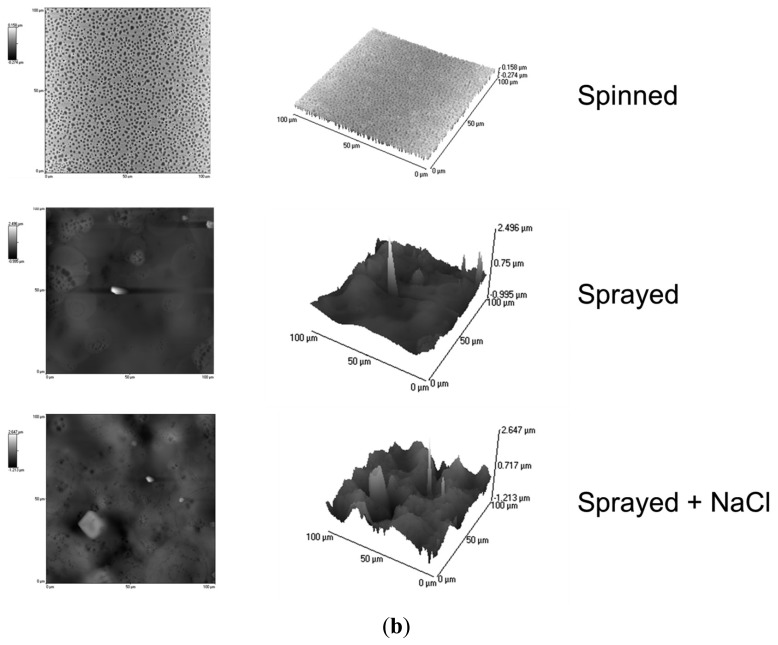
(**a**) SEM images of the different samples; (**b**) Left: AFM profile scans (one sample for each sample type) of a 100 × 100 μm area. The chromatic scales refer to sample heights. Right: 3D representations, corresponding to the 2D scans.

Concerning surface topography, first, a qualitative analysis was performed by analyzing the images obtained through a scanning electron microscope (SEM, EVO MA15 from Zeiss, equipped with LaB6 source and working at a 10 kV accelerating voltage). Significant differences can be observed between the various samples (Figure 10a).

More quantitative data concerning surface roughness were obtained by means of atomic force microscopy (AFM) analysis. AFM scans were performed by using an Innova Scanning Probe Microscope (Veeco). Measurements were performed in air, at room temperature, and operating in tapping mode, with oxide sharpened silicon probes (RTESPA-CP) at a resonant frequency of 300 kHz. AFM images were processed by means of a Gwyddion SPM software analysis tool.

We analyzed three samples for each deposition technique, and we acquired three 100 × 100 μm images for each sample. The spray technique coupled with salt leaching provided a structure with accentuated irregularities, with a roughness of 0.25 ± 0.013 μm. The spray deposition technique is also characterized by pores and irregularities, even if less pronounced: samples made by this technique were characterized by a roughness of 0.14 ± 0.006 μm. Spinned controls showed significantly lower roughness values: 0.05 ± 0.002 μm. These results are also confirmed by the 2D and 3D AFM images reported in Figure 10b.

## 6. System Validation

The system has been validated in terms of: (i) force monitoring in an *in vitro* controlled test bench; (ii) assessment of *in vitro* fibroblast proliferation on the polymeric coatings, provided with different surface topographies; and (iii) *ex vivo* assessment of wireless communication, carried out by considering a relevant environment, comparable to the *in vivo* one.

### 6.1. In Vitro Pressure Monitoring

**Figure 11.**
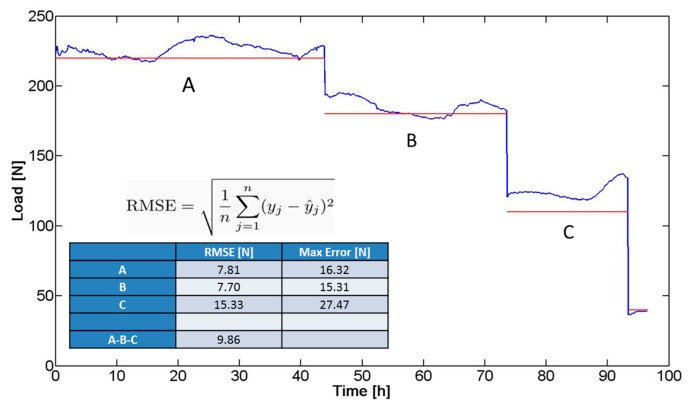
Force signal trend (Blue Line) and known compression force values applied with the mechanical testing system (Red Lines). The table shows the values of average errors found in every step (A–C) of the test. The errors are expressed as absolute values (N) and as a percentage of the ideal value. An average error of 8.85 N is derived.

In a preliminary validation phase, the system (bar and sensor) was subjected to a prolonged compression test (total duration: four days), using the mechanical testing system (*i.e.*, the same INSTRON 4464 previously detailed). The aim of the test was to simulate, *in vitro*, the forces and trends acting on the bar in terms of mechanical solicitations, once implanted. The imposed compression force was progressively lowered in the experimental course and kept constant at known force values for certain time intervals (Figure 11): 220 N (45 h, A), 180 N (28 h, B), and 110 N (20 h, C). The trend of the force signal (blue line in Figure 11) was compared with the ideal values (imposed values represented by the red line in Figure 11) and the overall root mean square error (RMSE) was calculated, resulting in equal to 9.86 N. The RMSE was calculated as the standard deviation of the differences between predicted values and observed values. Figure 11 shows the equation used to calculate the RMSE values: y is sensor output, ŷ is the pressure values predicted (applied pressure), and n is the numbers of observations. The maximum error revealed in Figure 11 was identified as the maximum discrepancy between the sensor output and the applied pressure value.

The factors contributing to the differences between the real trend and the ideal values can be due to: (i) inaccuracy of the compression machine; and (ii) approximations in the calibration curve. Although the response of the device did not exactly correspond to the imposed load, the trend of the signal acquired allowed to discriminate clearly different mechanical solicitations. This demonstrated the possibility to translate the device to a clinical scenario, characterized by the need of detecting trends of pressure exerted by the sternum on the bar, until reaching a plateau, which corresponds to the achievement of the deformity correction.

### 6.2. In Vitro Biological Responses

The increase of surface roughness, when nanometric, normally promotes cell proliferation [21]. Conversely, if the surface morphology is characterized by dimensionally larger irregularities, the effect obtained is the opposite, due to geometric impediments that limit cell ability to spread and divide [22]. To assess *in vitro* cell response to the different surface topographies, we seeded normal human dermal fibroblasts (nHDFs, purchased from Lonza, Cat. #CC-2511) on the different sample types, and we evaluated cell behavior at two different time-points, namely 24 h and 72 h after cell seeding. Samples were sterilized by means of UV irradiation for 45 min, then, nHDFs were seeded at a density of 5000 cells/cm^2^ on the sample surface. Samples were kept in standard culture medium, composed of 90% Dulbecco's Modified Eagle's Medium (DMEM, EuroClone, Milano, Italy), supplemented with 10% Fetal Bovine Serum (FBS, EuroClone, Milano, Italy), 100 IU/mL penicillin (EuroClone, Milano, Italy), 100 mg/mL streptomycin (EuroClone, Milano, Italy), and 2 mM L-glutamine (Sigma). During culture, the cells were maintained at 37 °C in a saturated humidity atmosphere containing 95% air and 5% CO_2_. At the two time-points, samples were rinsed with Phosphate Buffer Saline (PBS, EuroClone, Milano, Italy), then cell shape and number were qualitatively assessed by incubating them for 20 min with 2 μM AM-calcein (Molecular Probes, L3224): Live cells on the sample surface, thus, emitted green fluorescence. The samples were finally observed with an inverted fluorescent microscope (Eclipse Ti, FITC/TRITC filters, Nikon, Tokyo, Japan) equipped with a cooled Charge-Coupled Device (CCD) camera (DS-5MC USB2, Nikon, Tokyo, Japan) and with NIS elements imaging software.

**Figure 12.**
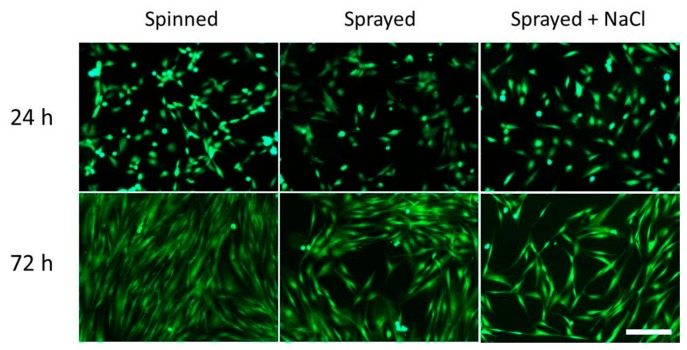
Fluorescence images (green = live cells) of nHDFs cultured on the different sample types, at the two time-points (24 h and 72 h). Scale bar = 100 μm.

The results of the validation tests (Figure 12) show that, 24 h after cell seeding, there is no substantial difference between the samples. This is mainly due to the material surface chemistry, which is the same for all the tested substrates (being based on PLA). Surface chemistry is the key factor, which determines the initial adhesion of cells to a substrate. However, after 72 h, the differences between the various samples become significant. On the samples characterized by a greater roughness (Sprayed + NaCl samples), nHDF proliferation was significantly reduced, thus, confirming the possibility to use this technique to reduce fibrotic response and tissue adherences to the implanted Nuss bar.

### 6.3. Ex Vivo Assessment of Wireless Communication

The effectiveness of the wireless communication between the RCE and the receiving system communication and conversion unit was assessed in an environment comparable to the *in vivo* one by using a rabbit and by performing *ex vivo* experiments. The RCE was inserted in the rabbit chest (dead rabbit, purchased at a butcher shop), then the tissues were sutured and the receiver was then progressively moved away from the RCE.

Despite the presence of soft tissues and bones, the communication was excellent up to distances of about two meters [23]. Such a communication distance can be considered more than acceptable, as it allows an efficient delivery of data during the measurement protocol (carried out by placing the receiver close to the patient's chest).

## 7. Conclusion and Future Works

This work proposes an advanced device for the treatment of PE. The study demonstrates how the reduction of the metal due to the integration of new components in the bar (*i.e.*, force sensor and polymeric coating) does not affect the structural stability of the device. The design of the electronics on board, and the remote station, are made to ensure the communication of the pressure data from the device to the user, as shown by the *in vitro* and *ex vivo* tests. The user exploits the HMI to acquire and save the data in a patient-specific database. The doctor will establish a monitoring protocol with an adequate number of controls operations during the time of implantation, also from a remote position. For future applications (*i.e.*, managing data by different sensing elements or selecting different program of data acquisition without the need of the magnetic-based trigger, also selecting power management modes for the microcontroller, to reduce the current consumption) a pseudokernel software platform will be exploited and the proposed software will be translated to an event-driven finite-state-machine code [24].

*In vitro* tests have also shown that a structured coating can decrease the fibrotic response of the tissues in response to the system, thus, facilitating the extraction of the bar. Compared with the current Nuss bar, the proposed system allows: (i) to monitor the advancement of the anatomical correction treatment without the use of X-rays; (ii) to study, patient-to-patient, the trend of correction in order to define therapeutic strategies customized for different classes of PE, age, gender, weight, and other factors; (iii) to study the force involved in the correction, usable for the design of a complete bioabsorbable bar; and (iv) to simplify the bar removal operation.

Further tests will be performed to evaluate the effectiveness of the sensorized Nuss bar in *in vivo* conditions.
